# Human Microbiota and Immunotherapy in Breast Cancer - A Review of Recent Developments

**DOI:** 10.3389/fonc.2021.815772

**Published:** 2022-01-28

**Authors:** Marina Vitorino, Susana Baptista de Almeida, Diogo Alpuim Costa, Ana Faria, Conceição Calhau, Sofia Azambuja Braga

**Affiliations:** ^1^ Medical Oncology Department, Hospital Professor Doutor Fernando Fonseca, Amadora, Portugal; ^2^ Breast Cancer Unit, CUF Oncologia, Lisbon, Portugal; ^3^ NOVA Medical School, Faculdade de Ciências Médicas, Lisbon, Portugal; ^4^ Faculdade de Medicina, Universidade de Lisboa, Lisbon, Portugal; ^5^ Comprehensive Health Research Centre (CHRC), NOVA Medical School, Faculdade de Ciências Médicas, Lisbon, Portugal; ^6^ CINTESIS – Center for Health Technology and Services Research, NOVA Medical School, Faculdade de Ciências Médicas, Lisbon, Portugal

**Keywords:** breast cancer, microbiota, microbiome, dysbiosis, pharmacomicrobiomics, treatment, immunotherapy

## Abstract

Breast cancer (BC) is the most common malignancy and the second cause of cancer-specific death in women from high-income countries. Infectious agents are the third most important risk factor for cancer incidence after tobacco and obesity. Dysbiosis emerged as a key player that may influence cancer development, treatment, and prognosis through diverse biological processes. Metastatic BC has a highly variable clinical course, and more recently, immune checkpoint inhibitors (ICIs) have become an emerging therapy in BC. Even with standardised treatment protocols, patients do not respond similarly, reflecting each individual´s heterogeneity, unique BC features, and tumour microenvironment. However, there is insufficient data regarding predictive factors of response to available treatments for BC. The microbiota could be a crucial piece of the puzzle to anticipate better individual BC risk and prognosis, pharmacokinetics, pharmacodynamics, and clinical efficacy. In recent years, it has been shown that gut microbiota may modulate cancer treatments’ efficacy and adverse effects, and it is also apparent that both cancer itself and anticancer therapies interact with gut microbiota bidirectionally. Moreover, it has been proposed that certain gut microbes may protect the host against inappropriate inflammation and modulate the immune response. Future clinical research will determine if microbiota may be a prognostic and predictive factor of response to ICI and/or its side effects. Also, modulation of microbiota can be used to improve outcomes in BC patients. In this review, we discuss the potential implications of metabolomics and pharmacomicrobiomics that might impact BC immunotherapy treatment.

## Introduction

The human gut microbiota contains ~3x10^13^ bacteria, most commensals (1). Microbiota plays a crucial role in balancing inflammation, infection and tolerance towards the commensal microbes and food antigens (2, 3). Furthermore, new evidence indicates that the microbiota influences oncogenesis and anticancer treatment outcomes by regulating local and systemic antitumour immunity (4).

Immunotherapy is a major emerging treatment for some haematological and solid tumours, including breast cancer (BC). Several BC clinical trials showed better outcomes with immune checkpoint inhibitors (ICI) than conventional chemotherapy. However, despite the promising data, the patients do not respond equally to immunotherapy treatments. Besides programmed death ligand-1 (PD-L1) expression, tumour-infiltrating lymphocytes (TILs), microsatellite instability (MMRd), and high tumour mutation burden (TMB), additional biomarkers for BC immunotherapy are still a significant unmet medical need (5–7).

Among these factors, the human microbiota could be a crucial piece of the puzzle to anticipate better individual BC predictive responses to ICI. More recently, studies have been showing the role of gut microbiota in modulating response and toxicity to ICI (8–10). This review highlights the relationship within the microbiota-host-breast cancer triad, exploring the potential implication of metabolomics and pharmacomicrobiomics that might impact BC immunotherapy treatment.

## Human Microbiota and Immune System

In a specific biosphere, the set composed of microorganisms, including bacteria, viruses, fungi, archaea, and protists, is designated by microbiota. The collective genome of these biological agents is called the microbiome. There are different microbiota ecosystems in the human body, such as the gastrointestinal tract, skin, vaginal mucosa, or the oral cavity, which account for trillions of microorganisms. The relationship between these ecological communities and the human body is ancient and evolved over time to benefit both parties simultaneously, thus achieving a symbiotic balance (11, 12).

The link between the host’s immune system and microbiota allows tolerance for commensal bacteria and the recognition of potentially infectious pathogenic microorganisms. The intestinal mucosa, below lamina propria, is composed of a layer that, among conjunctive tissue, possesses Peyer plates and immune cells, such as T and B lymphocytes and antigen-presenting cells (APC). This set of lymphoid tissue is named gut-associated lymphoid tissue (GALT), and it influences local and system immune responses (13). The communication of host and microbes is in charge of sensors, known as pattern recognition receptors (PRRs), like Toll-like receptors (TLR), expressed by intestinal epithelial cells and innate immune cells. These PRRs recognize microbe- or pathogen-associated molecular patterns (MAMPs or PAMPs). The microbiota recognition *via* these PRRs influences immune responses, both locally and systemically, and may induce the memory response, mediated by the transcriptional changes in genes or a specific locus and epigenetic rewiring of these cells upon the primary exposure (12, 14).

**Figure 1:**
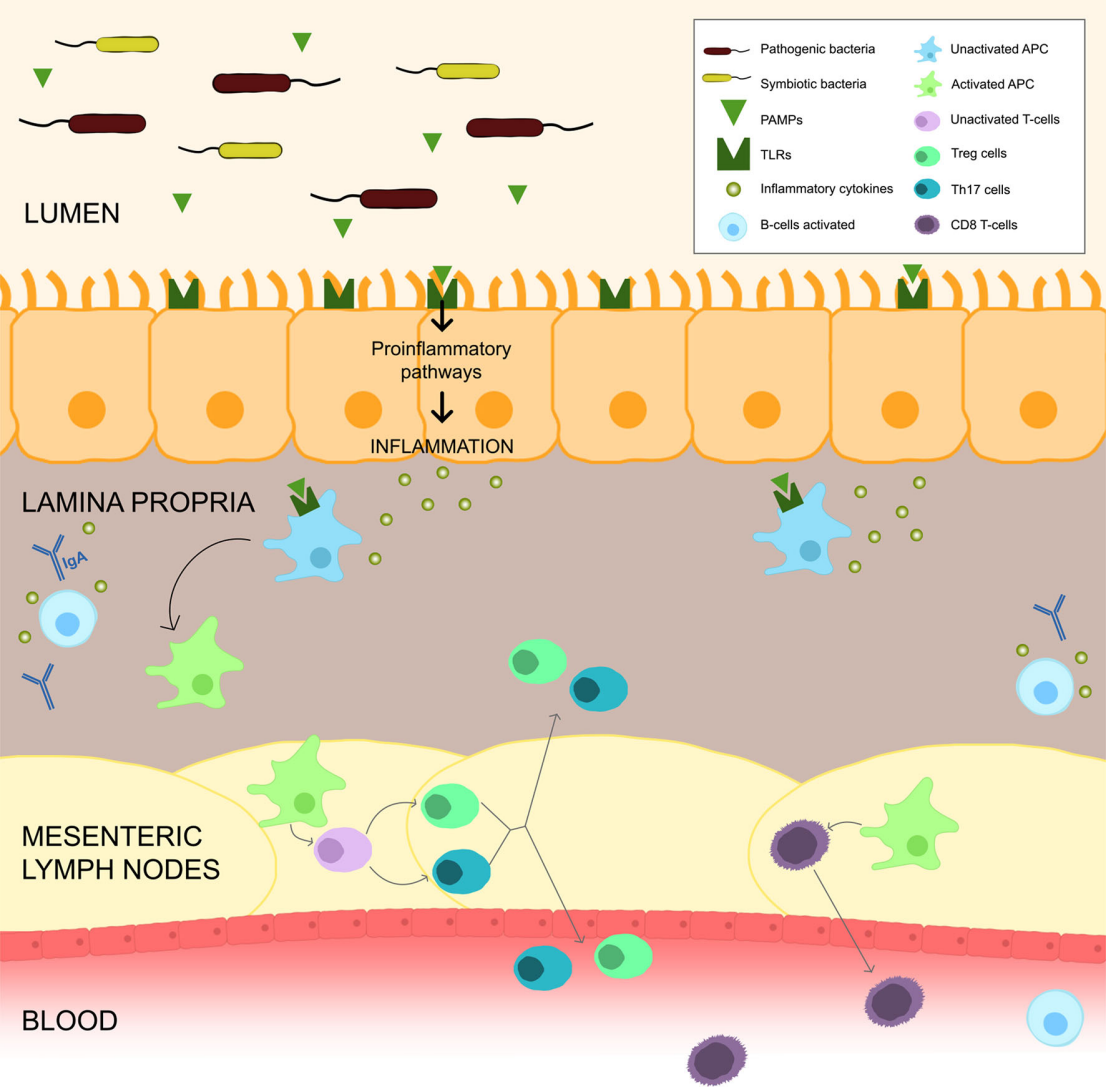
Gut Microbiota and Immune System. Gut bacteria, through PAMPs, can upregulate the TLRs and activate inflammatory pathways, which causes a release of cytokines leading to an inflammation milieu. PAMPs can also activate APC which migrate to the mesenteric lymph nodes to stimulate T and B cells. Activation of B cells to plasma cells allows the release of IgA into the lumen. APC activate CD4 T cells to differentiate into Tregs and Th17 cells, that can migrate back to the gut or enter systemic circulation and influence immunity in different sites. APC may also stimulate CD8 T cells into effector cells that migrate from the gut to periphery.

The bacterial metabolites directly interfere with the immune local cell’s actions, namely in the secretion of immunoglobulins (such as IgA), in the stimulation of lymphocytes differentiation into regulatory T-lymphocytes (Treg) and T helper 17 (Th17), in the production of immunomodulatory cytokines and even in the epigenetic regulation of histone deacetylase enzymes. The production of IgA by plasma cells improve immunity by blocking bacterial adherence to epithelial cells. In addition, the PAMPs derived from microbes promote the maturation of dendritic cells. These cells travel from the gut to mesenteric lymph nodes, where induce naïve CD4 T cells to differentiate into effector T cells (Tregs, Th17 cells). After maturation of these cells in the mesenteric lymph nodes, they can migrate back to the gut or enter systemic circulation and influence immunity in different sites. Circulating Th17 cells enhance antitumour immunity, protecting against bacterial and fungal infections, whereas circulating Tregs secrete anti-inflammatory cytokines. Activated by APC, these T cells can circulate systemically and allow an immune response against the same organism (12). The relationship between the microbiota and CD8 T cells remains poorly characterized, although recent studies showed that microbiota-mediated activation of these cells has implications in immunity and the response to cancer therapies (**Figure 1**). Some bacterial metabolites, like lipopolysaccharide (LPS), activate innate immune response by TLR pathway stimulation and then boosted antitumour CD8 T cells that migrate from the gut to the periphery (11, 15, 16).

Microbiota’s deregulation, with modifications in its functional composition and metabolic activity, is designated by dysbiosis and is linked to the development of inflammatory, auto-immune and malignant diseases. The changes in microbiota homeostasis leading to an imbalance in the symbiosis between the host and its organic habitat facilitate the loss of beneficial bacteria, overgrowth of potentially pathogenic microorganisms and loss of overall bacterial diversity. A break in the intestinal mucosa’s immunological barrier causes bacterial translocation, increased pro-inflammatory cytokines, and the recruitment of effector T-cells and neutrophils, generating a local and systemic inflammatory state (11, 17).

### Gut Microbiota and the Breast-Gut-Axis

The impairment of the normal functioning of gut microbiota in maintaining host wellness may deregulate the microbial-derived products or metabolites, causing several other disorders on local or distant organs, including in the tissue breast (10, 18). In this context, some microorganisms seem to interfere with host cell proliferation and apoptosis, tissue inflammation, cell invasion, immune system function, gene expression, oncogenic signalling, mutagenesis, angiogenesis, and hormonal and detoxification pathways (10, 19, 20). In addition, human microbiota’s composition also influences drug disposition, action and toxicity, including of ICI (10, 21–23).

Concerning the links between human microbiota and BC, some risk modulating metabolites are already known, such as oestrogens, active phytoestrogens, short-chain fatty acid (SCFA), lithocholic acid (LCA) and cadaverine. Oestrogen formation in gut microbiota is mainly due to β-glucuronidase (BGUS) activity, which is a part of the enzymatic complex of specific intestinal bacteria. The metabolism of these BGUS-producing bacteria leads to deconjugation of xenobiotics and sexual hormone oestrogens and to an increase of oestrogens reabsorption into the systemic circulation that may increase the risk of hormone-dependent BC in women (10, 20, 24). Furthermore, several studies have shown differences in local and gut microbiota between BC patients and healthy controls (10, 25). On the other hand, other metabolites are linked to a protective or risk-reducing factor for BC development, including phytoestrogens, LCA and cadaverine (10). The manipulation of microbiota to select certain types of microorganisms, with the support of specific diets, prebiotics, probiotics or symbiotics, postbiotics, antimicrobial agents or even through fecal microbiota transplantation (FMT), is being studied and pondered, either as a prophylactic approach or as a therapeutic use for BC (10, 24).

The mechanism by which gut bacteria can promote BC is also through chronic inflammation, which is associated with tumour development. Gut bacteria, through PAMPs, can upregulate the TLR and activate NF-kB, which is an important inflammation regulator associated with cancer. The activation of NF-kB causes the release of several cytokines, like IL-6, IL-12, IL-17 and IL-18 and the tumour necrosis factor-alpha (TNF-alpha), leading to persistent inflammation in the tumour microenvironment. The PAMPs are recognized by innate-immune system cells and are essential components for pathogens such as the bacterial LPS, flagellin, lipoteic acid, peptidoglycans and unmethylated CpG oligodeoxynucleotides (26). In addition, secondary metabolites released by intestinal bacteria along with pro-inflammatory molecules that reach the liver *via* portal vein may promote carcinogenesis. Butyrate, an intestinal microbial metabolite, can directly enhance the antitumour cytotoxic CD8 T cell response by modulating the ID2-dependent manner of the IL-12 signalling pathway (27).

The gut microbiome also contributes to epigenetic deregulation, which can interact with the tumour. The microorganisms can produce low molecular weight bioactive substances such as folates, short-chain fatty acids and biotin, which can participate in epigenetic processes, including altering substrates used for methylation or synthesising the complexes that change the action of epigenetic enzymes (28).

## Breast Cancer, Microbiota and Immunotherapy

BC is the most common malignancy, and the incidence and the number of survivors continues to increase, with most developed countries reporting 85-90% five-year survival rates. However, patients with BC show different outcomes, according to different molecular profiles. Currently, four molecular subtypes of BC with prognostic and therapeutic relevance are well established: luminal A-like subtype, with high expression of oestrogen (ER) and progesterone (PR) receptors and low cell proliferation index; luminal B-like subtype with high expression of ER and PR and high cell proliferation index; HER2 overexpressing subtype and triple negative (TNBC) subtype (ER/PR and HER2 negative) (29, 30). Furthermore, depending on histological subtype and stage at diagnosis, the prognoses are different, with the luminal A-like and TNBC subtypes having the best and worst prognoses, respectively.

The most relevant BC risk factors are advanced age, exposure to endogenous and exogenous oestrogens, high breast density, history of atypical hyperplasia, personal or family history of breast disease, genetic predisposition and environmental factors (31). In addition, current evidence points to other clues for a complementary mechanism of non-hereditary risk of BC. Infectious agents are known to be the third most important risk factor after tobacco and obesity, contributing to 15–20% of cancer incidence. Gut microbiota is, as mentioned previously, an emerging field of research that is being associated with cancer through direct and indirect interference in diverse biological processes: host cell proliferation and death, immune system function, chronic inflammation, oncogenic signalling, hormonal and detoxification pathways (10, 32, 33).

Most BC patients are diagnosed in initial stages when the goal of treatment is to cure. In early and locally advanced BC, a multimodal approach is frequently used, incorporating surgery, radiotherapy and systemic therapy. The primary goals of treatment are to prolong survival and ameliorate the quality of life (10, 31).

Immunotherapy has become a forefront treatment of patients with specific malignancies. ICI utilise the immune system to exert an antitumour effect, suppressing the interaction of T-lymphocyte inhibitory receptors with their ligands on malignant cells, thereby re-stimulating the T-lymphocyte-mediated immune response against tumour-associated antigens (5, 7). BC is not traditionally considered a highly immunogenic tumour compared with other malignancies, such as lung cancer or melanoma, which have the highest rate of TMB. Although, recent data have shown immunotherapy benefits, mainly in the TNBC subtype. Usually, this BC subgroup of patients has a dismal prognosis, with worse survival and early relapse rates (34). KEYNOTE-012, phase Ib trial, investigated pembrolizumab monotherapy in previously treated TNBC patients and revealed an overall response rate (ORR) of 18.5% and a median time to response of 17.9 weeks (35). A phase II study using pembrolizumab (KEYNOTE-086) as first-line therapy for metastatic TNBC showed a safety profile and antitumour efficacy with an ORR of 23% (36). Other phases I trials, NCT01375842 and JAVELIN, evaluated the use of atezolizumab and avelumab and observed ORR of 10% and 5.2%, respectively (37, 38). A combination of immunotherapy with chemotherapy was also intensively investigated. Atezolizumab combined with nab-paclitaxel was tested in patients with metastatic TNBC, and the ORR was 67% in the first line, 25% in the second line, and 29% in the third or further lines (39). The phase III trial IMpassion 130 investigated the combination of atezolizumab plus nab-paclitaxel in untreated metastatic TNBC patients. In the intention-to-treat population (ITT) analysis, there was a progression-free survival (PFS) benefit in the combination arm (chemotherapy with atezolizumab), with 7.2 months vs 5.5 months. This benefit was most prominent in the PD-L1 positive population analysis, with 7.5 months vs 5.0 months. In the ITT population analysis of overall survival (OS), the benefit in the experimental arm was not statistically significant (21.3 months vs 17.6 months HR 0.84, 95% CI 0.69 to 1.02; P=0.08), but in the PD-L1 positive population, there was an increase in OS (25.0 months vs 15.5 months, HR 0.62; 95% CI, 0.45 to 0.86) (40). The ENHANCE-1/KEYNOTE-150 phase Ib/II trial evaluated eribulin combined with pembrolizumab, in which the ORR was higher in PD-L1-positive BC patients (30.6% vs 22.4%) (41). The KEYNOTE-355 trial evaluated the combination of pembrolizumab with chemotherapy, and patients were stratified according to PD-L1 value (combined positive score (CPS) ≥ 1, CPS≥ 10). In the CPS≥10 population, there was a significant PFS benefit in the pembrolizumab arm, 5.6 months vs 9.7 months (HR for progression or death, 0-65, 95% CI 0-49-0-86; one-sided p=0-0012) (42).

### Links Between Microbiota and Immunotherapy

Treatment with immunotherapy has revolutionised cancer treatment over the past few years. However, not all patients will experience a favourable response to treatment. Thereby, predictive markers are of utmost importance for the physician to know whether the ICIs will benefit the patient.

Even with standardised treatment protocols, patients do not respond similarly, reflecting each individual´s heterogeneity, unique BC features, and tumour microenvironment (10). There is insufficient data regarding predictive factors of response to immunotherapy treatments for BC. HER-2+ and TNBC are also more likely to express PD-L1 in the tumour microenvironment than luminal BC (43, 44). Higher levels of TILs and CD8+ T-cell/Treg ratio at diagnosis predict benefit from adjuvant and neoadjuvant chemotherapy (45, 46). Some tumours that harbour TILs and express PD-L1 are more likely to respond to ICI, suggesting this may also be the case for BC (47). In recent years, it has been shown that gut microbiota may modulate cancer treatments’ efficacy and toxicity. On the other hand, it is also apparent that both cancer itself and anticancer therapies interact with gut microbiota bidirectionally. The pharmacomicrobiomics studies may support the potential use of gut microbiota analysis to predict patients’ response to treatments, allowing a more personalised approach based on the microbiota-host-cancer triad (48–50).

The response and toxicity to ICI can be affected by gut microbiota (**Table 1**). In studies with mouse models, it was shown that specific microbes influence responses to this type of treatment differently, and a cause-effect relationship was established between the presence of a certain bacterial species within the intestinal microbiota and a favourable therapeutic outcome for the immune-based treatments (8, 9). A better response to anti-PD-(L)1 therapy was observed in mice with specific species of microbiota (e.g., *Akkermansia muciniphila*, *Bifidobacterium longem*, *Collinsella aerofaciens*, *Faecalibacterium prausnitzii*) (9, 10). In addition, recent data reported that OS and PFS rates were significantly higher in patients who had not received antibiotics before and during ICI treatment compared to those who had received (10, 56). Germ-free or antibiotic-treated mice received FMTs from patients’ responders to ICIs and were inoculated with tumour cell lines two weeks after FMT and treated with ICIs targeting PD-1 or PD-L1. FMT from responders were enriched in *Akkermansia muciniphila*, *Bifidobacterium longum*, *Collinsela aerofaciens* and/or *Faecalibacterium* spp. The efficacy observed in mice undergoing FMT with responder faeces was associated with enhanced priming of CD45+ and CD8+ T cells in the intestine. Thus, antibiotics may pose some risk for dysbiosis due to the lack of specificity in the type of bacteria eliminated by their repeated use (54).

**Table 1:** Clinical studies with association between gut microbiota and efficacy/toxicity of immune checkpoint inhibitors.

Reference	Study population	Results	
		Favourable microbiota	Unfavourable microbiota
Dubin et al. (51) Nature Communications 2016	Metastatic melanoma patients who received ipilimumab	Lower risk of anti-CTLA-4-induced colitis: • *Bacteroidaceae*, *Barnesiellaceae, Rikenellaceae*	–
Chaput et al. (52) Annals of Oncology 2017	Metastatic melanoma patients who received ipilimumab	Lower risk of anti-CTLA-4-induced colitis: • *Bacteroides* spp. associated with less anti-CTLA-4-induced colitis	Higher risk of anti-CTLA-4-induced colitis: • *Faecalibacterium prausnitzii*, *Gemmiger formicilis*, butyrate producing bacterium L2-21
Gopalakrishnan et al. (11) Science 2018	Metastatic melanoma who received PD-1 inhibitors	Higher clinical response: • > gut bacterial diversity • *Faecalibacterium prausnitzii*	Lower clinical response: • < gut bacterial diversity • *Anaerotruncus colihominis, Bacteroides thetaiotaomicron, Escherichia coli*
Matson et al. (53) Science 2018	Metastatic melanoma who received PD-1 inhibitor	Higher clinical response: • *Akkermansia muciniphila, Bifidobacterium adolescentis, Bifidobacterium longum, Collinsella aerofaciens, Enterococcus faecium, Klebsiella pneumoniae, Lactobacillus* spp.*, Parabacteroides merdae, Veillonella parvula*	Lower clinical response: • *Roseburia intestinalis, Ruminococcus obeum*
Routy et al. (54) Science 2018	Metastatic urothelial carcinoma, NSCLC, and RCC who received PD-1/PD-L1 inhibitors	Higher clinical response: • ↑ *Akkermansia muciniphila, Alistipes* spp.*, Eubacterium* spp., *Ruminococcus* spp. • ↓ *Bifidobacterium adolescentis*, *Bifidobacterium longum*, *Parabacteroides distasonis*	–
Vetizou et al. (8) Science 2015	Advanced melanoma and NSCLC who received ipilimumab	Higher clinical response: • *B. fragilis, B. thetaiotaomicron*	–
Frankel et al. (55) Neoplasia 2017	Metastatic melanoma patients who received ICI	Higher clinical response: • *Bacteroides caccae*, *Bacteroides thetaiotamicro, Dorea formicogenerans, Faecalibacterium prausnitzii*, *Holdemania filiformis*	–

CTLA-4, cytotoxic T-lymphocyte-associated antigen 4; ICI, immune checkpoint inhibitors; NSCLC, non-small cell lung cancer; PD-L1, programmed death-ligand 1; RCC, renal cell carcinoma.

On the other hand, gut microbiota may also influence ICI toxicity (**Table 1**). Some studies have shown that patients with specific bacteria (e.g., *Bacteroidaceae*, *Barnesiellaceae*, *Rikenellaceae*) have a higher risk of immune-mediated toxicity. Evidence suggests that most colitis-associated phylotypes were related to Firmicutes (relatives of *Faecalibacterium prausnitzii* and *Gemmiger formicilis*), whereas no colitis was assigned to Bacteroidetes (52). In 2016 a prospective study with 34 patients analysed the intestinal microbiota with the subsequent development of ICI-induced colitis. Bacteroidetes phylum is enriched in colitis-resistant patients and is consistent with an immunomodulatory role of these commensal bacteria (51).

## Conclusion

A plethora of immunotherapy options is now part of treatment armamentarium of several malignancies, including BC. Unfortunately, despite this remarkable success, only a minority of BC patients respond to ICI and there is insufficient data regarding predictive factors of response.

In recent years, it has been shown that both cancer itself and anticancer therapies interact with gut microbiota bidirectionally. Thus, the pharmacomicrobiomics studies may support the potential use of gut microbiota analysis to predict patients’ response to ICI, allowing a more personalised and precision medicine in oncology. Also, microbiota manipulation can be used to improve treatment outcomes in BC patients. However, further studies are necessary to validate microbiota analysis and modulation as part of the ‘real world’ BC clinical practice.

## Author Contributions

MV, SB, and DA contributed to the conception and design of the review. MV and SB wrote the first draft of the manuscript. All authors contributed to manuscript revision, read, and approved the submitted version

## Conflict of Interest

The authors declare that the research was conducted in the absence of any commercial or financial relationships that could be construed as a potential conflict of interest.

## Publisher’s Note

All claims expressed in this article are solely those of the authors and do not necessarily represent those of their affiliated organizations, or those of the publisher, the editors and the reviewers. Any product that may be evaluated in this article, or claim that may be made by its manufacturer, is not guaranteed or endorsed by the publisher.
